# Supplementary material for: Mettl15-Mettl17 modulates the transition from early to late pre-mitoribosome
Source: bioRxiv. 2025 Jan 24:2024.12.18.629302. Preprint. [Version 2] doi: 10.1101/2024.12.18.629302 (PMC11785013; doi:10.1101/2024.12.18.629302)
Supplement: 1 [file NIHPP2024.12.18.629302V2-supplement-1.pdf]

bioRxiv preprint doi: https://doi.org/10.1101/2024.12.18.629302; this version posted January 24, 2025. The copyright holder for this preprint (which was not certified by peer review) is the author/funder, who has granted bioRxiv a license to display the preprint in perpetuity. It is made available under aCC-BY 4.0 International license.

Table S1. Newly identified features of the *T. brucei* pre-mtSSU

Protein	***Previous name or chain ID***	***Newly described feature(s)***
mt-SAF38	chains UY, Ue	newly identified assembly factor
Mettl15	mt-SAF14	homolog of Mettl15 (RsmH in *E. coli*)<br>cofactor SAM
Mettl17	mt-SAF1	homolog of Mettl17<br>cofactor SAM<br>iron-sulphur cluster Fe_4S_4
RbfA	mt-SAF18	homolog of RbfA
mt-SAF16<br>mt-SAF19<br>mt-SAF25	-	homologs of *Saccharomyces cerevisiae* Mam33[59] (Uniprot ID P40513), human p32 (Q07021[60]) and *Chlamydomonas reinhardtii* mtSSU protein mS105[61] (A0A2K3DAY3)
mS53	-	residues 63-84 modeled
mt-SAF5	-	residues 560-596 modeled
mt-SAF10	-	residues 4-6 modeled
mt-SAF11	-	residues 148-156 modeled
rRNA	-	several regions modeled or adjusted (see Methods)
mt-SAF10<br>mt-SAF22<br>mt-SAF27	chains UB, UC, UD, UF, UI, UJ, UM, UN	ligand acetyl coenzyme A

bioRxiv preprint doi: https://doi.org/10.1101/2024.12.18.629302; this version posted January 24, 2025. The copyright holder for this preprint (which was not certified by peer review) is the author/funder, who has granted bioRxiv a license to display the preprint in perpetuity. It is made available under aCC-BY 4.0 International license.

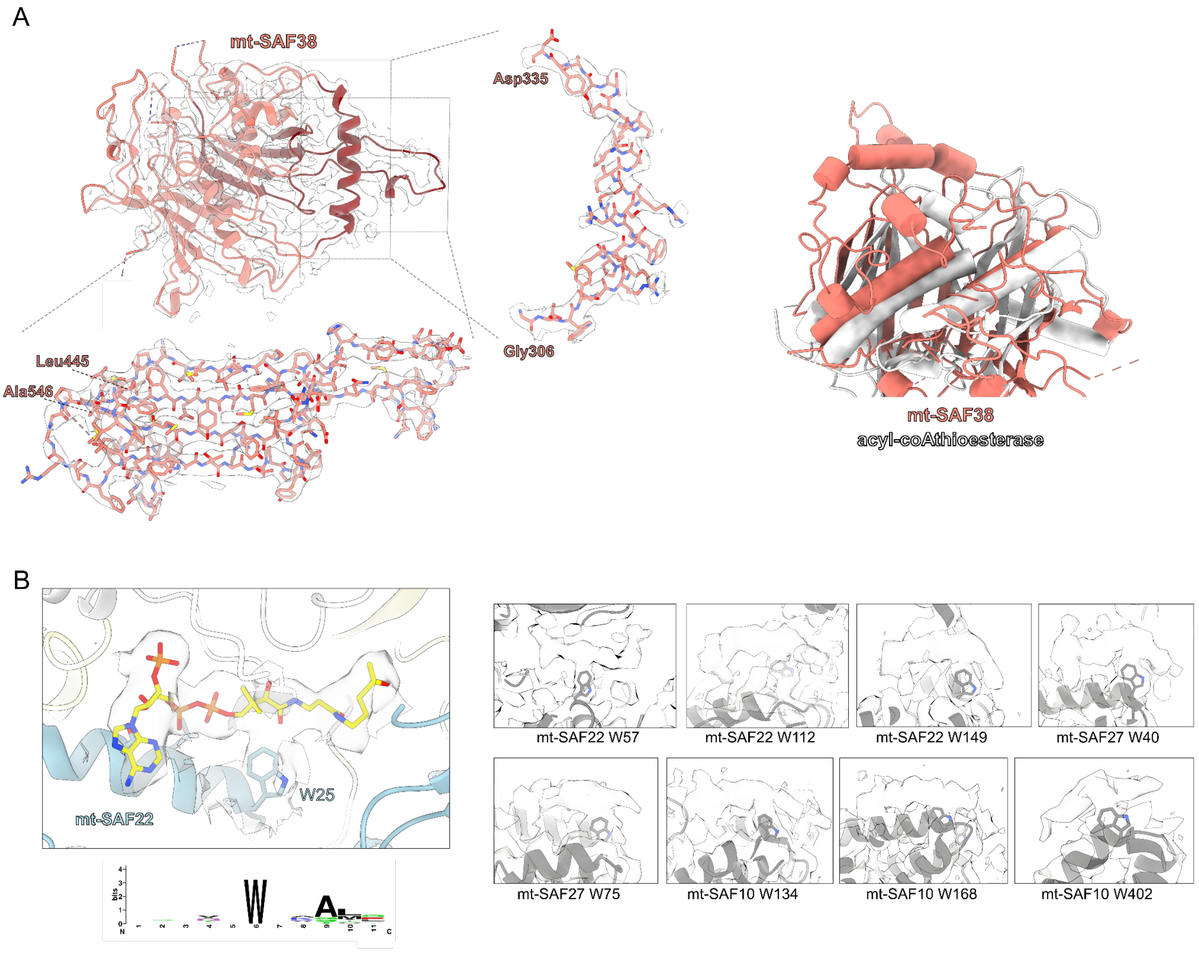

Figure S1. New features in the map. (A) The density and model of the newly identified mt-SAF38 with close-up views showing how residues fit the density. Right, mt-SAF38 (dark red) superposed with mouse acyl-coA thioesterase (PDB 5ZV3). **(B)** Acetyl-CoA placed into a density associated with tryptophan 25 of mt-SAF22 and other examples of the hammerhead shaped densities. Sequence logo of acetyl-CoA binding regions, showing the conserved tryptophan, was created using WebLogo[75].

bioRxiv preprint doi: https://doi.org/10.1101/2024.12.18.629302; this version posted January 24, 2025. The copyright holder for this preprint (which was not certified by peer review) is the author/funder, who has granted bioRxiv a license to display the preprint in perpetuity. It is made available under aCC-BY 4.0 International license.

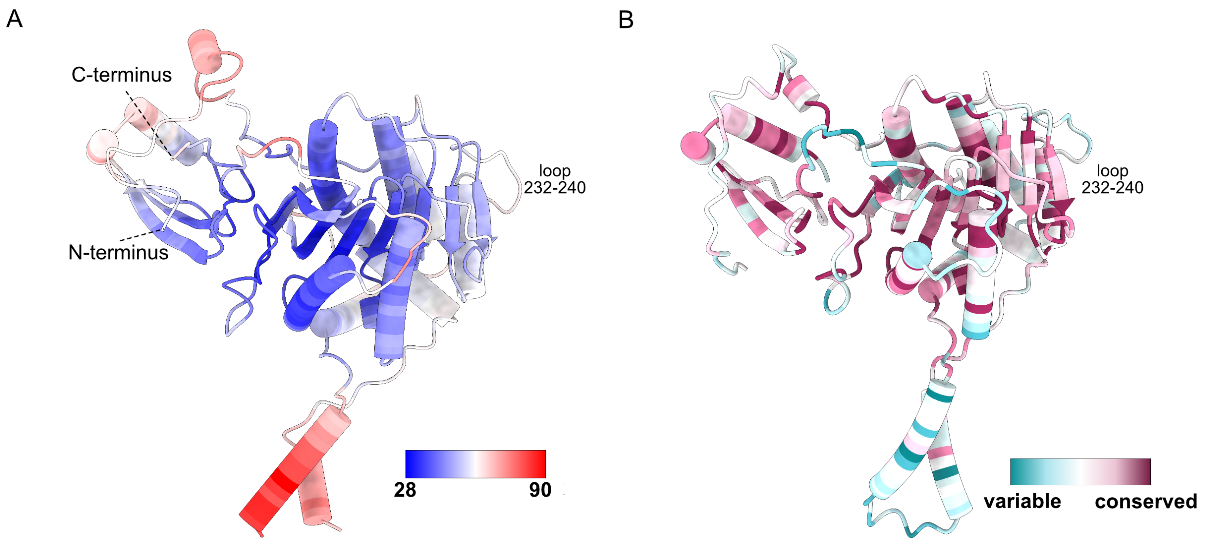

Figure S2: Flexibility and conservation of Mettl17. (A) PDB ID 8CST colored by B-factor. Increased flexibility of the loop 232-240 is evident by a higher B-factor. **(B)** The conservation coloring profile calculated by ConSurf repository[76] mapped onto the model.

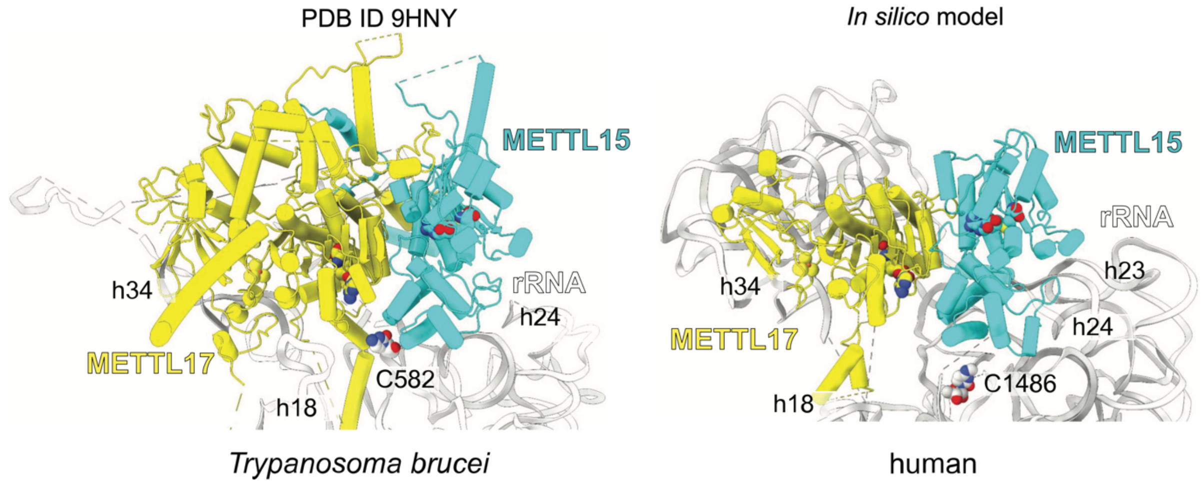

Figure S3: Comparison of the Mettl15-Mettl17 heterodimer in *T. brucei* and corresponding *in silico* model of the human mitoribosome. Human early-stage pre-mitoribosomal model PDB ID 8CST was aligned onto *T. brucei* Mettl17, and Mettl15 was modelled based on the trypanosomal template with no clashes. The position of Mettl15 in the created *in silico* model is compatible with the experimental data.

bioRxiv preprint doi: https://doi.org/10.1101/2024.12.18.629302; this version posted January 24, 2025. The copyright holder for this preprint (which was not certified by peer review) is the author/funder, who has granted bioRxiv a license to display the preprint in perpetuity. It is made available under aCC-BY 4.0 International license.

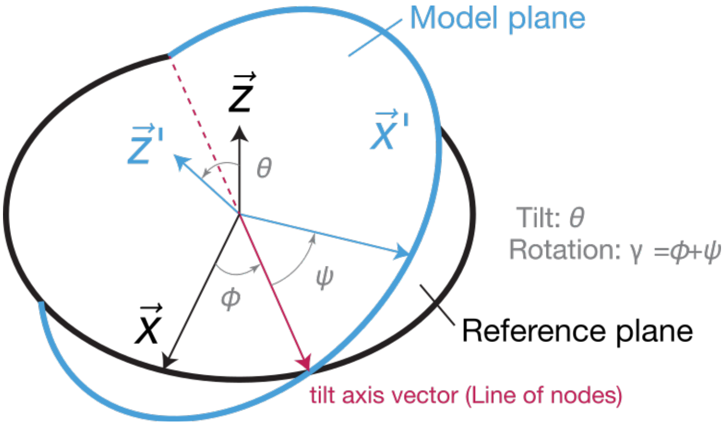

Figure S4: Description of Euler angles used to analyze molecular dynamics simulation. The angles were calculated by comparing the vectors of the model plane $\vec{z}', \vec{x}'$ with the corresponding vectors of the reference plane $\vec{z}, \vec{x}$. Rotation is defined be angle $\gamma = \phi + \psi$, while tilt is defined by angle θ around the tilting axis (line of nodes).
